# Cancer is an RNA disease: DNA is not destiny, RNA determines leukemia fate

**DOI:** 10.1038/s41375-026-03041-x

**Published:** 2026-07-13

**Authors:** Inge van der Werf, Catriona Helen Macleod Jamieson

**Affiliations:** Sanford Stem Cell Institute, Division of Regenerative Medicine, Department of Medicine, University of California San Diego, La Jolla, CA USA

**Keywords:** Haematological cancer, Haematopoietic stem cells

The increasing incidence of myeloid cancers with age is commonly attributed to the accumulation of somatic pathologic variants throughout life. For example, the frequency of clonal hematopoiesis wherein variant hematopoietic stem cell clones increases with age [1]. However, several questions remain unresolved. Not all variants underlying CH result in haematological abnormalities or progress to leukaemia. Also, the same variant can be associated with different cancer phenotypes and trajectories. Further, there is a distinct peak in leukemia incidence during infancy despite the limited time to acquire somatic pathogenic variants. These data suggest somatic variants alone do not fully explain leukemia development. Prior data indicate that haematopoietic stem cell behavior and intrinsic and extrinsic factors shape disease evolution [2]. However, the molecular process(es) linking aging to selective clonal outgrowth is uncertain. We propose RNA dysregulation may be the missing layer. Somatic variants may identify risk, whilst changes at the RNA level may influence trajectory and determine leukemia fate.

## RNA is not simply the messenger

RNA is often portrayed as an intermediate between DNA and protein. In reality, RNA is dynamically and tightly regulated, thereby determining how genetic information is interpreted in a cell type- and context-specific manner. Post-transcriptional processes, including RNA splicing, editing and methylation control transcript localization, translation, stability and decay, processes which shape cell identity and function [3]. Among these processes, RNA splicing most clearly shows RNA is not merely a messenger but a central regulator of gene output and biological complexity. Splicing takes place in > 90 percent of our genes. This process uses alternative splice sites, which produce distinct transcripts that code for different protein isoforms that exhibit unique structural and functional properties. The importance of this became especially apparent in the early 2000s when data from Celera Genomics and the Human Genome Project indicated humans have only 20,000–25,000 protein-coding genes, far fewer than anticipated. This limitation underscores how alternative splicing enables relatively few genes to generate remarkable molecular and cellular diversity. Notably, RNA is involved in the regulation of alternative splicing (e.g., small nuclear RNAs U2 and U6), emphasizing that RNA is not simply a messenger but also a catalyst [3].

In stem cells, alternative splicing is now recognized as a key regulator of gene expression, in which small perturbations can alter self-renewal and lineage commitment [4]. Yet, RNA splicing represents only one dimension of RNA regulation. RNA molecules are also modified and regulated through processes such as adenosine-to-inosine editing, m^6^A methylation, or by binding of non-coding RNAs, including microRNAs [5–7]. These layers influence transcript stability, localization, and translational efficiency. Importantly, many of these mechanisms change with aging or upon cellular stress, suggesting they may cooperate with somatic mutations to determine whether a clone remains dormant, expands, or transforms.

## Aging compromises transcriptomic integrity

Hematopoietic stem cells (HSCs) sustain lifelong blood production through their capacity for self-renewal and multi-lineage differentiation. However, with aging, HSC *fitness* declines. Increasing data indicate this decline reflects altered transcriptional programs associated with aging and altered RNA processing, the latter characterized by increased transcriptional variability, reduced coordination between transcription and RNA processing and altered RNA splicing and editing patterns [2, 8]. A recent study reported that aging is associated with increased RNA polymerase II elongation speed [9]. Because splice-site recognition requires high precision, even subtle changes in transcriptional kinetics can impair splice site recognition, generate aberrant isoforms and/or introduce frameshifts which alter protein function. Increased polymerase speed is associated with widespread alterations in splicing patterns and transcript processing, suggesting a progressive decline in transcriptional and post-transcriptional integrity and increased transcriptomic instability during aging [9].

Age-associated RNA changes are not random. Aging drives selective remodeling of gene expression programs, which converges on key hallmarks of aging [8]. Comparative analyses of cells from young and old donors have identified transcriptional alterations in inflammatory, mitochondrial and proteostatic pathways [8]. These data indicate coordinated shifts in cell programs rather than uniform, global disruption of RNA processing. Analyses of HSCs indicate distinct age-associated splice isoform signatures, including altered splicing of transcription factors involved in myeloid lineage specification, including ETV3 and CEBPB, as well as inflammation-responsive regulators like NFIL3 and IRF1 [10]. Thus, aging reshapes RNA output at the transcriptional and post-transcriptional levels, influencing pathways central to stem cell function, differentiation and inflammation.

Aging is increasingly defined by epigenetic clocks based largely on DNA methylation dynamics, which indicates that age-related change involves dynamic regulatory processes beyond DNA variants [8]. Moreover, age-associated loss of epigenetic silencing may derepress repetitive elements such as long interspersed nuclear element-1 (LINE-1) and human endogenous retroviruses (HERVs), features of stem cell aging [2]. These responses may increase reliance on RNA surveillance pathways whilst simultaneously driving chronic inflammatory signaling, promoting interferon-responsive adenosine deaminase acting on RNA 1 (ADAR1)-mediated RNA editing and increased apolipoprotein B mRNA editing enzyme catalytic polypeptide-like 3 (APOBEC3) activity implicated in the expansion of hematopoietic stem and progenitor cells [11, 12]. Together, these data suggest the notion that clonal hematopoiesis results solely from acquiring variants is incomplete.

## Rethinking clonal hematopoiesis

Age-related clonal hematopoiesis is associated with increased risks of leukemia, cardiovascular disease and death. It is typically viewed as a variant-driven process in which somatic pathologic variants in epigenetic regulators (e.g., DNMT3A, TET2, ASXL1, IDH1 and 2), spliceosome components (e.g., SRSF2, SF3B1, U2AF1, ZRSR2) and/or JAK2 confer a competitive growth advantage enabling variant hematopoietic stem cells to outcompete their normal counterparts [13].

In vivo experiments, largely performed in model organisms, often interpret clonal expansion as a consequence of enhanced stem cell self-renewal or growth advantage [13]. However, more recent studies in humans indicate clones arise already early in life and need another driver variant to expand [14–16]. Analyses of 4536 CHIP carriers showed substantial heterogeneity in clonal fitness [15]. Based on an extensive model inferring clonal expansion rate from a single time point, variants in SRSF2, SF3B1 and U2AF1 splicing genes and JAK2V617F were among the fastest growing, consistent with previous estimates of fitness from longitudinal sequencing of samples with clonal hematopoiesis, implying the driver variant is the determinant of clonal expansion [15]. What other factors drive the positive selection of these clones is unknown.

One possibility is that aging progressively impairs hematopoietic function by age-associated inflammation, niche dysfunction and RNA dysregulation. In this setting, variant clones may expand not because they are intrinsically stronger but because they are comparatively more resilient than aged normal stem cells. Selection would then reflect declining hematopoietic stem cell fitness rather than only an intrinsic superiority of variant clones. Notably, downregulation of the RNA-binding protein MSI2 has been identified as a mechanism that limits clonal hematopoiesis, suggesting post-transcriptional regulation can constrain clonal expansion and influence clonal dynamics through clonal competition [17]. Admittedly, this reflects only one dimension as RNA dys-regulation can also directly drive clonal expansion and leukemia evolution.

## RNA dysregulation across leukemias

Somatic variants in spliceosome genes like SF3B1, SRSF2, U2AF1 and ZRSR2, which induce widespread aberrant splicing, are identified in several cancers with a strikingly high frequency amongst adults with myeloid leukemias [18]. Yet, even without these spliceosome variants, splicing dysregulation is seen and is characterized by differential exon use and the emergence of new splice isoforms. Similar patterns are seen in leukemias in children, where splicing factor mutations and age-related CH-associated mutations are uncommon, but RNA splicing dysregulation persists [19, 20].

Beyond splicing alterations, RNA editing is increasingly implicated in hematologic cancers and is closely linked to inflammatory and interferon-driven stress responses. Increased expression of ADAR1, an RNA A-to-I editing enzyme, is associated with progression from chronic to blast phase chronic myeloid leukemia (CML), suggesting a potential role in disease evolution [11]. More recently, ADAR1-mediated A-to-I RNA editing has been reported in children with acute myeloid leukemia and to promote progenitor reprogramming in T-cell acute lymphoblastic leukemia (T-ALL) [19, 21]. ADAR1 activity is also implicated in myelo-proliferative neoplasms (MPNs) and clonal hematopoietic disorders with a risk of leukemia transformation, supporting a role in leukemia progression [22]. Importantly, APOBEC3C, a C-to-U RNA editing enzyme, is up-regulated in myelofibrosis at risk for transformation to acute myeloid leukemia [12]. These associations suggest RNA editing programs may underlie leukemia transformation in diverse settings.

Similarly, RNA methylation and microRNA-mediated regulation are implicated in modulating transcript stability, translation, oncogenic signaling, and stem cell programs in the context of leukemia. These processes are interconnected, illustrated by RNA editing of splice sites, hyper-editing of cell cycle regulatory microRNAs and 3’UTR binding sites of cancer suppressor-associated microRNAs [12, 23–25]. RNA dysregulation is a hallmark of leukemia, which may represent a parallel layer of regulation preceding clonal hematopoiesis, reinforce clonal selection or expansion, or enhance downstream functional consequences of associated somatic variants.

## Conclusion

**Fig. 1:**
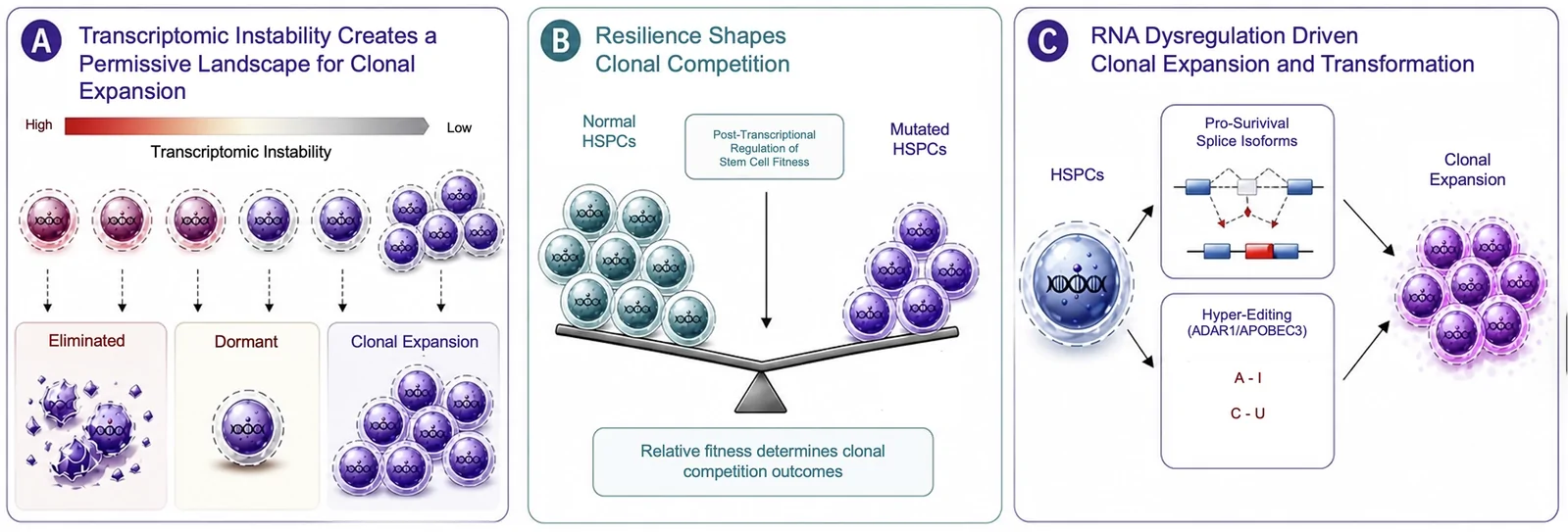
RNA determines leukemic fate. **A** Aging-associated decline in transcriptomic integrity and increased transcriptomic instability create a permissive landscape wherein hematopoietic stem and progenitor cell (HSPC) clones expand based on relative resilience rather than intrinsic fitness. **B** Post-transcriptional regulation can also constrain clonal expansion and influence clonal dynamics by resilience-based clonal competition. **C** RNA dysregulation can drive clonal expansion through pro-survival splicing programs and aberrant RNA editing (e.g., ADAR/APOBEC activity). In this framework, genetic variants promote diversity, but RNA may influence trajectory and determine leukemic fate (Generated using Manus AI).

We suggest RNA is not simply a passive messenger of DNA-encoded information. We propose that hematopoietic stem cells exist within an RNA landscape defined by transcriptomic integrity and adaptability. Key variables include splicing accuracy, RNA editing balance, translational capacity, inflammatory signaling and stress responses. In this framework, cancer may develop not only from the accumulation of 1 or more pathologic *driver* variants but also when RNA regulation is disordered, especially in the context of aging. RNA dysregulation may also drive clonal hematopoiesis by promoting survival via splicing and hyper-editing such that DNA variants *seed* clones, but RNA regulation determines expansion and/ or transformation (Fig. 1).
